# Endometrial Carcinoma, Grossing and Processing Issues: Recommendations of the International Society of Gynecologic Pathologists

**DOI:** 10.1097/PGP.0000000000000552

**Published:** 2018-12-14

**Authors:** Anais Malpica, Elizabeth D. Euscher, Jonathan L. Hecht, Rouba Ali-Fehmi, Charles M. Quick, Naveena Singh, Lars-Christian Horn, Isabel Alvarado-Cabrero, Xavier Matias-Guiu, Lynn Hirschowitz, Máire Duggan, Jaume Ordi, Vinita Parkash, Yoshiki Mikami, M. Ruhul Quddus, Richard Zaino, Annette Staebler, Charles Zaloudek, W. Glenn McCluggage, Esther Oliva

**Affiliations:** Department of Pathology, The University of Texas MD Anderson Cancer Center, Houston, Texas (A.M., E.D.E.); Department of Pathology, Beth Israel Deaconess Medical Center and Harvard Medical School (J.L.H.); Department of Pathology, Massachusetts General Hospital and Harvard Medical School (E.O.), Boston, Massachusetts; Department of Pathology, Wayne State University, Detroit, Michigan (R.A.F.); Department of Pathology, University of Arkansas for Medical Sciences, Little Rock, Arkansas (C.M.Q.); Department of Cellular Pathology, Barts Health NHS Trust, London (N.S.); Department of Cellular Pathology, Birmingham Women’s NHS Trust, Birmingham (L.H.), UK; Division of Gynecologic, Breast and Perinatal Pathology, University Hospital, Leipzig (L.C.H.); Institute of Pathology, University Hospital of Tübingen, Tübingen (A.S.), Germany; Department of Pathology, Hospital de Oncología Siglo XXI, Mexico City, Mexico City, Mexico (I.A.C.); Department of Pathology, Hospital Universitari de Bellvitge and Hospital Universitari de Arnau de Vilanova, Idibell, Irblleida, and Universitat de Lleida, Ciberonc (X.M.G.); Department of Pathology, Hospital Clinic of Barcelona, Universitat de Barcelona, Barcelona (J.O.), Spain; Department of Pathology and Laboratory Medicine, Cumming School of Medicine, University of Calgary, Alberta, Canada (M.D.); Department of Pathology, Yale University School of Medicine, New Haven, Connecticut (V.P.); Department of Diagnostic Pathology, Kumamoto University Hospital, Kumamoto, Japan (Y.M.); Department of Pathology, Women and Infants Hospital and The Warren Alpert Medical School of Brown University, Providence, Rhode Island (M.R.Q.); Division of Anatomic Pathology, Hershey Medical Center, Pennsylvania State University, Hershey, Pennsylvania (R.Z.); Department of Pathology, University of California, San Francisco, San Francisco, California (C.Z.); Department of Pathology, Belfast Health and Social Care Trust, Belfast, UK (W.G.M.)

**Keywords:** Endometrial carcinoma, Gross examination, Macroscopic examination, Processing, Pathology, Tumor size, Staging, Lymph nodes, Sentinel lymph nodes

## Abstract

Endometrial cancer is the most common gynecologic neoplasm in developed countries; however, updated universal guidelines are currently not available to handle specimens obtained during the surgical treatment of patients affected by this disease. This article presents recommendations on how to gross and submit sections for microscopic examination of hysterectomy specimens and other tissues removed during the surgical management of endometrial cancer such as salpingo-oophorectomy, omentectomy, and lymph node dissection—including sentinel lymph nodes. In addition, the intraoperative assessment of some of these specimens is addressed. These recommendations are based on a review of the literature, grossing manuals from various institutions, and a collaborative effort by a subgroup of the Endometrial Cancer Task Force of the International Society of Gynecological Pathologists. The aim of these recommendations is to standardize the processing of endometrial cancer specimens which is vital for adequate pathological reporting and will ultimately improve our understanding of this disease.

Endometrial cancer is the sixth most common malignant neoplasm in women worldwide and is the most common gynecologic malignancy in developed countries 1. In the United States, the American Cancer Society estimates ∼63,230 new cases of endometrial cancer and 11,350 deaths due to this disease in 2018 2. Although no updated universal guidelines are currently available on how to gross the surgical specimens obtained in cases of endometrial cancer, it is widely accepted that a thorough gross/macroscopic examination will optimize the acquisition of information required for proper diagnosis, staging, treatment, and prognosis. This article presents the recommendations to handle and gross such specimens as proposed by the members of the Endometrial Cancer Task Force of the International Society of Gynecological Pathologists. These recommendations cover not only hysterectomy specimens, including those obtained prophylactically in patients at risk of developing this disease, but also salpingo-oophorectomy and omentectomy specimens, as well as sentinel and non–sentinel lymph nodes (SLN). This work is based on a review of the literature, grossing manuals from various institutions, and a collaborative effort by a subgroup of the above-mentioned task force. These recommendations will assist in standardizing the processing of these specimens, which is critical not only for an accurate pathology report, but also to improve our knowledge of this disease.

## RECOMMENDATIONS

### General

All pathology reports should include a detailed section code/block key on which the origin/designation of all tissue blocks should be recorded. This information is particularly important should there be a need for internal or external review as reviewers need to be clear about the origin of each tissue block in order to provide an informed specialist opinion. Recording the origin/designation of all tissue blocks also facilitates retrieval of blocks for immunohistochemical or molecular analysis, research studies or clinical trials.

## HYSTERECTOMY SPECIMEN HANDLING

### General Rule of Gross/Macroscopic Examination

**FIG. 1:**
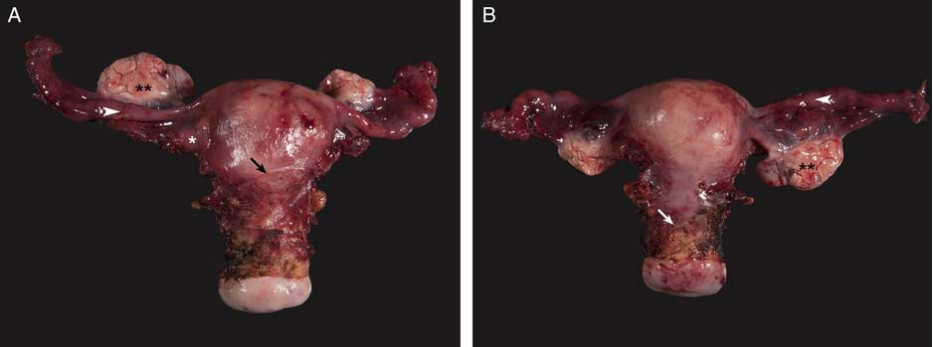
Orientation of hysterectomy specimen using anatomical landmarks, peritoneal reflection is higher anteriorly (arrow) and the sequence of structures in the adnexal region is round ligament (*), fallopian tube (arrowhead) and ovary (**) (A), peritoneal reflection is lower posteriorly (arrow) and the sequence of structures in the adnexal region is ovary (**), fallopian tube (arrowhead) and round ligament (B), the latter is not visualized in this photograph.

Orient the specimen, that is identify the anterior and posterior walls of the uterus using anatomic landmarks such as the peritoneal reflection and the round ligament/ovaries (Figs. 1A, B). Document all organs/structures received and record their measurements and gross appearance.

## WHEN SHOULD THE UTERUS BE OPENED?

### Recommendation

Uteri should be opened immediately upon receipt in the pathology laboratory and placed in formalin within an hour of opening whenever possible.

The purpose of opening the uterus immediately is to prevent autolysis, which is very common in hysterectomy specimens, and to avoid potential preanalytical issues when ordering immunohistochemical or molecular studies 3–5. Although most of the grossing manuals used in academic institutions in North America do not provide specific timelines, some include specific instructions such as opening and fixation within 1 h of receipt in the pathology laboratory, documentation of cold ischemic interval and interval in formalin, and prompt procurement of fresh tissue for banking and/or investigational protocol purposes 6.

## SHOULD THE PATHOLOGY PERSONNEL MANAGE REQUESTS FOR FRESH TISSUE FOR STUDIES?

### Recommendation

The pathology laboratory personnel and/or pathologists should manage the requests for fresh tissue for banking and/or investigational protocols and this task should be completed as soon as the specimen is received in the pathology laboratory.

Procurement of fresh tissue for banking or research protocols should be done as soon as possible as prolonged ischemia affects tissue quality for investigational purposes. Tissue procurement for research should not compromise pathologic evaluation 6–10. Therefore, a pathologist should be consulted in questionable cases. In addition, the tissue procured for research should be available for diagnostic purposes if needed.

## SHOULD THE PERITONEAL AND/OR NONPERITONEAL SURFACES BE INKED?

### Recommendation

Inking of peritoneal and/or nonperitoneal surfaces is recommended in hysterectomy specimens and is mandatory in radical hysterectomy specimens in which parametrium and vaginal cuff are present.

**FIG. 2:**
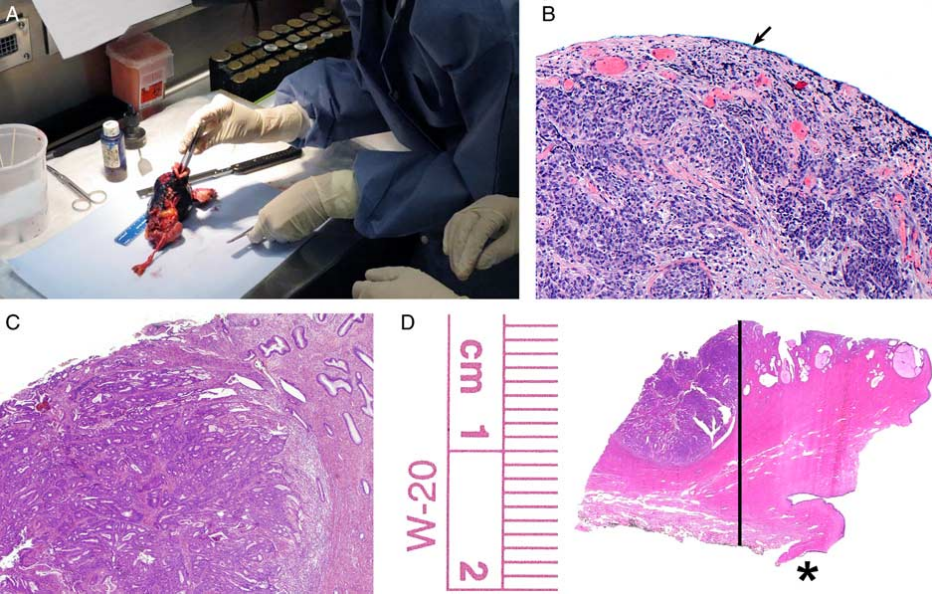
Inking the anterior and posterior uterine serosal surfaces with extension of the ink to the ectocervix or vaginal cuff margin (A), helps to confirm the presence of tumor in the uterine serosa (arrow) (B), and in cases with cervical stromal involvement (C) the measurement of the relationship of the stromal invasion to the full thickness of the cervical wall (line), and the proper identification of the ectocervical or vaginal cuff margin (*) (D).

Serosal inking can aid in specimen orientation and in confirming the presence of tumor at the uterine serosal surface if present (Figs. 2A, B). In addition, inking the peritoneal and nonperitoneal surfaces and extending the ink all the way to the ectocervical/vaginal cuff margin in cases where the tumor has invaded the cervical stroma is useful to provide an accurate measurement of the depth of cervical stromal invasion relative to the full thickness of the cervical wall and the status of the ectocervical/vaginal cuff margin (Figs. 2C, D). Of note, measurement of the depth of cervical stromal invasion and its relationship with the full thickness of the cervical wall are not included in the College of American Pathologists (CAP) protocol for endometrial cancer 11, but they are often required by radiation oncologists to delineate treatment recommendations 12. The practice of inking is variably addressed in the multiple grossing manuals reviewed. Some manuals do not mention it at all while others recommend inking as follows: (1) serosal/nonserosal surfaces for orientation purposes, (2) inking serosal abnormalities only to confirm tumor at the serosal surface, and (3) inking only of the parametrium and vaginal cuff if present. Overall, inking is helpful, but it is important to blot the inked surfaces right after applying the ink to avoid ink displacement and the potential misinterpretation of this finding.

## SHOULD THE UTERINE WEIGHT BE INCLUDED IN THE REPORT?

### Recommendation

The need to include the uterine weight in the pathology report is geographically dependent. This parameter has to be provided in the United States because the Current Procedural Terminology (CPT) code required for reimbursement purposes changes according to a uterine weight of 250 g (i.e. CPT code 58570 is used for a uterus with a weight of ≤250 g while CPT code 58572 is used for the same specimen if the uterus weighs >250 g) 13–15.

Although uterine weight could be related to surgical outcome as larger uteri may be associated with increased duration of surgery or risks of surgical complications 16–18, the main reason to include uterine weight in the pathology report is to ensure proper reimbursement for the procedure when this is performed in the United States. In addition, this information is also required for the case list submitted by candidates applying to certification by the American Board of Obstetrics and Gynecology in the United States 19.

## HOW SHOULD THE UTERUS BE OPENED?

### Recommendation

The uterus should be opened along the lateral uterine walls (3 and 9 o’clock).

**FIG. 3:**
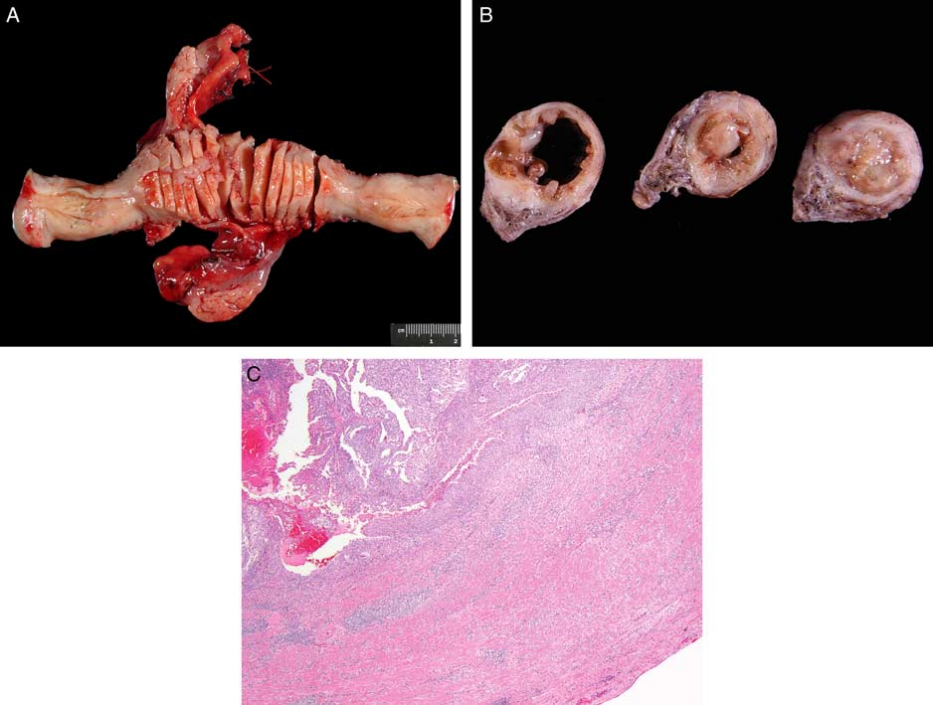
Opening the uterus at 3 and 9 o’clock provides a maximum exposure of the endometrial surface in a flat plane to better visualize and measure an endometrial tumor (A), the cornua can contain tumor (B) and this should not be mistaken for myometrial invasion (C).

The above method provides maximum exposure of the endometrial surface in a flat plane which allows better visualization and measurement of the tumor (Fig. 3A). Of note, the lateral uterine walls contain the cornua and the pathologist should be aware that tumor involving the lumen of the proximal portion of the fallopian tube can be interpreted as myoinvasive carcinoma (Figs. 3B, C). All grossing manuals examined for this review, in which this procedure is described, recommend opening the uterus along the lateral walls 6.

## SHOULD THE TUMOR ALWAYS BE MEASURED IN 3 DIMENSIONS?

### Recommendation

At least the largest dimension of the tumor must be provided, although providing 3 dimensions of a neoplasm is often considered “best practice” and represents standard pathology practice.

**FIG. 4:**
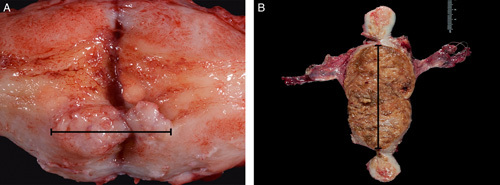
Measurement of endometrial carcinoma, small polypoid tumor (A), a tumor that involves the anterior and posterior walls in a continuum, carpet-like fashion should be measured accordingly (B).

Currently, there is some controversy with regards to the impact of tumor size on patient outcome; nevertheless, tumors exceeding various established thresholds have been associated with increased stage of disease and/or risk of recurrence 20–27. Therefore, at a minimum, the largest dimension of the tumor should be reported (Figs. 4A, B). The other dimensions can be recorded, but are not required. Some manuals specify the need to record the 3 dimensions of a tumor; however, this is not a uniform approach as requirements for measurements are not specifically outlined in several of the manuals reviewed while others require 2 or only 1 tumor dimension 6. The Royal College of Pathologists Dataset for Histopathological Reporting of Endometrial Cancer in the United Kingdom 28 recommends only recording the maximum tumor dimension while the current CAP checklist includes only the maximum tumor dimension as an optional element which is not required for accreditation 11. Another important issue regarding tumor size is that this parameter has a pivotal role in one of the algorithms used to triage patients intraoperatively for lymph node dissection in the setting of low-risk endometrial carcinoma. The so-called “Mayo algorithm” is based on an association between tumor size and risk of lymph node metastases 21,22. According to this algorithm, patients with endometrioid adenocarcinoma, FIGO grades 1 or 2, measuring ≤2 cm and with ≤50% myometrial invasion are spared of pelvic lymph node dissection as the risk of lymph node metastases is <0.3% while patients with similar tumors measuring >2 cm and ≤50% myometrial invasion undergo a dissection of pelvic lymph nodes as the risk of pelvic lymph node metastases increases to 10% 21.

The controversy between tumor size and disease outcome can be summarized as follows: (1) some studies have shown an association between tumor size and risk of lymph node metastases, but tumor size has not been found to be an independent predictive factor when depth of myometrial invasion and lymphovascular invasion are included in multivariate analysis 23,24; (2) some studies have demonstrated an association between tumor size, either ≥3.5 or >5 cm, and recurrence risk and/or prognosis in otherwise low-risk cancers 25,26; (3) other studies have not shown an association between tumor size and prognosis 24,27.

## HOW SHOULD THE UTERUS BE SECTIONED (HORIZONTAL/TRANSVERSE OR LONGITUDINALLY)?

### Recommendation

Horizontal/transverse sectioning is recommended.

**FIG. 5:**
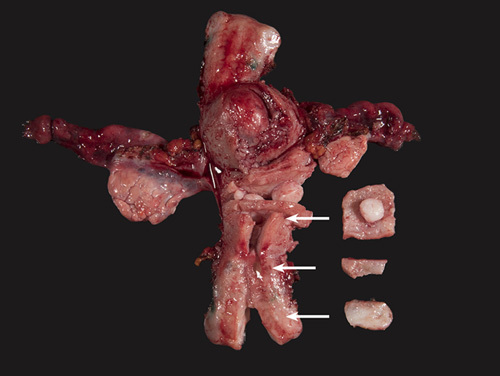
Cross-sections of the uterine wall at the level of the corpus (top arrow), longitudinal sections at the level of the lower uterine segment (middle arrow) and cervix (bottom arrow).

All manuals reviewed, except one, suggest the use of horizontal/transverse sectioning (left to right) from the lower uterine segment to the fundus. However, vertical sectioning is advisable to demonstrate the lower uterine segment in conjunction with upper cervix 6 (Fig. 5).

## SHOULD THE TUMOR BE SUBMITTED ENTIRELY FOR HISTOLOGIC EXAMINATION? IF NOT, HOW SHOULD THE NUMBER OF SECTIONS BE DETERMINED?

### Recommendation

It is not necessary to submit an endometrial tumor in its entirety for microscopic examination. Sampling one section per centimeter of the largest tumor dimension (as is done with tumors at other anatomic sites) will suffice.

Grossing manuals provide a wide range of recommendations; however, none advocates submitting the entire tumor unless it measures ≤3 cm. Several manuals advocate one section per centimeter of largest tumor dimension 6. Also, a proposal for submitting at least 4 blocks of tumor has been presented 28. Of note, there are no data explicitly addressing the value of extensive histologic examination of an endometrial carcinoma. Although the risk of missing a high-grade (serous, clear cell, undifferentiated or neuroendocrine) carcinoma component in association with an endometrioid carcinoma exists, these cases are uncommon 29.

## HOW MANY SECTIONS DO YOU TAKE IF YOU DO NOT SEE CARCINOMA ON GROSS EXAMINATION, IF THERE IS A HISTORY OF ATYPICAL ENDOMETRIAL HYPERPLASIA/ENDOMETRIOID INTRAEPITHELIAL NEOPLASIA (EIN) OR IF UNSUSPECTED ATYPICAL ENDOMETRIAL HYPERPLASIA/ EIN OR ENDOMETRIAL CARCINOMA IS DETECTED IN THE REPRESENTATIVE SECTIONS OF A HYSTERECTOMY OBTAINED FOR OTHER REASONS?

### Recommendation

The entire endometrium and adjacent inner myometrium should be submitted for microscopic examination in the setting of a preoperative endometrial sampling demonstrating malignancy and no visible lesion on gross examination or if there is a history of atypical endometrial hyperplasia/EIN. The same applies to hysterectomy specimens that have been obtained for other reasons (leiomyomas, adenomyosis, etc.) with no gross endometrial lesion and endometrial carcinoma or atypical endometrial hyperplasia EIN detected on microscopic examination of the initial representative sections. Sections of residual myometrium should be placed in sequential order, either in folded paper towels or gauze, in a formalin filled container in case that the examination of additional myometrial tissue is needed to determine an accurate depth of myometrial invasion if any. In addition, cornual blocks need to be submitted in cases of biopsy proven carcinoma, but no gross endometrial tumor.

Most reviewed manuals recommend submitting the entire endometrium when a preoperative diagnosis of malignancy has been rendered and no gross lesion is seen in the hysterectomy, or in cases of atypical endometrial hyperplasia/EIN 6. In addition, the Royal College of Pathologists of the United Kingdom recommends the submission of cornual blocks in cases of biopsy proven carcinoma, but no visible endometrial tumor on gross examination 28. Of interest, the intraoperative evaluation of 1 random full thickness section of the uterine wall from hysterectomies obtained for endometrial carcinoma and no gross lesion demonstrated the presence of microscopic tumor only in 3 (15%) of 20 cases with a final diagnosis of cancer 30.

## HOW MANY SECTIONS SHOULD INCLUDE THE FULL THICKNESS OF THE MYOMETRIUM TO DETERMINE THE MAXIMUM DEPTH OF INVASION?

### Recommendation

At least, one full thickness section of the uterine wall—including serosa, is required to show the deepest point of myometrial invasion. In uteri with a relatively thin wall which allows the submission of a full thickness section in a single cassette, it is possible to submit all sections containing tumor as full thickness sections.

**FIG. 6:**
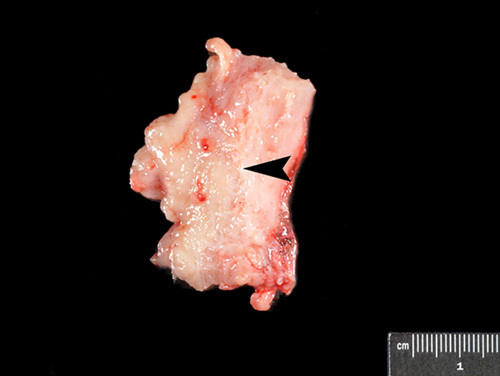
Cross-section of the uterine wall demonstrating deepest point of myometrial invasion on gross examination (arrowhead).

The manuals reviewed offered a wide range of recommendations, from at least one full thickness section to all submitted sections containing tumor to be of full thickness 6,28. In ideal circumstances, a single full thickness section submitted after a careful gross examination would be sufficient to determine the deepest point of myometrial invasion (Fig. 6). However, myometrial invasion assessment may be complicated in certain circumstances such as cases with adenomyosis involved by carcinoma, cases with peculiar patterns of myometrial invasion like “adenoma malignum-like,” “single cells,” “microcystic, elongated, and fragmented” 31 or if an underlying leiomyoma is present distorting the uterine wall architecture. It should be noted that the examination of the myometrium is important not only for assessment of depth of tumor invasion, but also to identify the unusual infiltrative patterns mentioned above and to identify and quantify lymphovascular invasion. Regarding the assessment of the deepest myometrial invasion in frozen section, several studies have examined this issue and the number of sections used has ranged from 1 to 5 32–37. Depth of myometrial invasion is an independent predictor of both lymph node metastases and prognosis 38 and is part of the FIGO staging system 39. In cases where the gross examination does not allow the submission of the deepest point of invasion with confidence or in problematic cases such as those above, processing several full thickness sections of the uterine wall may be necessary in order to render an accurate assessment.

## IF ADENOMYOSIS IS SUSPECTED, HOW MANY SECTIONS SHOULD SAMPLE THE FULL THICKNESS OF THE MYOMETRIUM TO DETERMINE THE MAXIMUM DEPTH OF INVASION?

### Recommendation

The number of sections submitted should not be altered in the context of adenomyosis. However, in cases where the assessment of myometrial invasion is difficult because of tumor involving adenomyosis taking additional sections of the uterine wall may be useful.

None of the manuals reviewed addresses the measurement of myometrial invasion in the context of adenomyosis 6.

## SHOULD THE TUMOR/NONTUMOR INTERFACE BE SAMPLED?

### Recommendation

Whenever possible, the tumor/nontumor interface should be submitted for microscopic examination. This facilitates the measurement of the depth of myometrial invasion and the identification of precursor lesions.

Some of the manuals reviewed specifically indicate the need to sample the tumor/nontumor interface 6.

## HOW MUCH NON-NEOPLASTIC ENDOMETRIUM SHOULD BE SUBMITTED?

### Recommendation

At least one representative section of non-neoplastic endometrium should be submitted for microscopic examination. In addition, any grossly identified endometrial lesions separate from the tumor should be submitted.

There is no universal recommendation about the number of sections of grossly unremarkable endometrium to be submitted. Some manuals recommend submitting one or 2 full thickness sections of uninvolved endomyometrium 6.

## HOW MANY SECTIONS OF ENDOMETRIUM AND ADNEXAL STRUCTURES SHOULD BE TAKEN IF THE PATIENT IS KNOWN TO HAVE LYNCH SYNDROME?

### Recommendation

All gross endometrial abnormalities need to be submitted for microscopic examination. In the absence of a gross lesion, the endometrium should be submitted in toto. Macroscopic examination and sampling should be performed after fixation, in an orderly manner, for example sequentially from superior to inferior or vice versa. Sections should include the endomyometrial interface. The lower uterine segment should be submitted in toto with longitudinal sections including the endocervical junction. Unremarkable ovaries and fallopian tubes should be submitted in toto although there is no evidence at the present time to support this practice.

Pathologists should be informed of a history of Lynch syndrome to handle the specimen properly 40–42. The demonstration of clinically occult endometrial carcinomas, some being microscopic, underscores the need for in toto submission of the endometrium in the absence of macroscopic lesions. Sections should include the endomyometrial interface, but the excess myometrium can be trimmed 40,43. Unremarkable ovaries and fallopian tubes should also be submitted in toto until enough experience is accumulated to support or modify this practice 40,43,44.

Women with Lynch syndrome are at increased risk of malignancies, with colorectal, endometrial and ovarian cancer being the most frequent 45–48. Endometrial cancer is the most common extracolonic tumor in these patients 47,49. Endometrial cancer cumulative incidence at 70 years is consistently high for patients with MLH1 (34%), MSH2 (51%), MSH6 (49%), and PMS2 (24%) germline mutations, and is frequently the first malignancy diagnosed in women with Lynch syndrome 50. Ovarian cancer is the second most common extracolonic neoplasia in women with Lynch syndrome with a cumulative lifetime risk ranging from 6% to 14% 51–56. Prophylactic hysterectomy and bilateral salpingo-oophorectomy has been advocated as a cost-effective measure that significantly reduces the risk of gynecologic cancer in Lynch syndrome patients. This procedure is currently included in the National Comprehensive Cancer Network (NCCN) guidelines for the management of patients with Lynch syndrome who are either postmenopausal or who have completed childbearing 41,57–61. The implementation of this procedure has been increasing progressively 41,62. Endometrial findings in prophylactic hysterectomy specimens in these patients include atypical hyperplasia and small and low grade endometrioid carcinomas 40,43,44,63. The finding of small incidental endometrial carcinomas in these specimens emphasizes the need to submit the entire endometrium for microscopic examination 40,43,44,63. Regarding the need to submit in toto unremarkable fallopian tubes and ovaries for microscopic examination, using the SEE-FIM (Sectioning and Extensively Examining the FIMbriated End) protocol, there is no current evidence to support this practice 40,43,44,63; however, as experience with these prophylactic specimens is still limited, we recommend submitting unremarkable adnexal structures entirely for microscopic examination as it has been proposed by a group of investigators 43.

## HOW MANY SECTIONS OF THE LOWER UTERINE SEGMENT SHOULD BE TAKEN? SHOULD THEY BE TAKEN HORIZONTALLY OR VERTICALLY?

### Recommendation

A minimum of 2 sections (1 anterior, 1 posterior) should be submitted from the lower uterine segment. Longitudinal sections are encouraged as they demonstrate the relationship with the upper endocervix.

Most manuals reviewed advocate the use of longitudinal sections of the lower uterine segment (1 anterior/1 posterior) 6.

## SHOULD THE PARAMETRIAL TISSUE/PARAMETRIUM BE SAMPLED BEFORE OPENING THE UTERUS TO AVOID CARRYOVER? HOW MUCH OF THE TISSUE SHOULD BE SUBMITTED?

### Recommendation

Parametrial tissue/parametrium should be sampled before opening the uterus as this approach minimizes the chance of finding carryovers. All of the parametrial tissue/parametrium should be submitted for histologic examination. After inking the parametrial margin, the parametrial tissue/parametrium should be blocked in sequential slices. If macroscopic tumor is seen in the parametrial tissue/parametrium, the most proximal parametrial section should include the adjacent outer portion of the cervical wall.

Very few of the manuals address this issue, and, when mentioned, recommend removing the parametrial tissue/parametrium before opening the uterus 6. Radical hysterectomies may be undertaken in cases of endometrial cancer with an epicenter in the lower uterine segment or with prominent cervical involvement, and, as expected, this type of specimen will contain parametrium. In contrast, simple hysterectomies, which are the specimens obtained for most endometrial carcinomas, do not usually contain parametrium; however, a very small amount of parametrial tissue may sometimes be included.

## SHOULD THE CERVIX BE AMPUTATED OR LEFT ATTACHED TO THE CORPUS?

### Recommendation

The cervix should be left attached to the corpus during the gross examination of a hysterectomy specimen obtained for endometrial carcinoma.

No practice manual recommends amputation of the cervix 6. Amputation could potentially interfere in the pathologic assessment of the tumor and the relationship with the upper endocervix, which can be problematic even at the microscopic level 64.

## HOW MANY SECTIONS OF THE CERVIX SHOULD BE TAKEN IF GROSSLY NORMAL?

### Recommendation

At least 2 full thickness sections (1 anterior and 1 posterior) should be submitted from a grossly unremarkable cervix. In cases of high-grade endometrial carcinoma or when the tumor is grossly close to the cervix, submitting additional sections might be considered. It is important to submit full thickness sections of the uterine cervix because the presence of tumor in the cervical stroma triggers measurement of its depth of invasion in relation to the thickness of the cervical wall.

All manuals reviewed except one advocate the submission of 2 representative sections as indicated above 6. The Royal College of Pathologists makes a similar recommendation 28. One study found no value in submitting more than the standard 2 sections of cervix (1 anterior and 1 posterior) in hysterectomies for endometrial cancer without a gross lesion in the cervix 65. However, a more recent publication found that submitting the 2 standard sections of grossly unremarkable cervix missed 24% of endometrial carcinomas involving the cervix. Of note, in the latter publication, the patient population was high-risk including patients with serous carcinomas, FIGO grade 3 endometrioid carcinomas, carcinosarcomas, and carcinomas with lymphovascular invasion 66.

## HOW MANY SECTIONS OF CERVIX SHOULD BE TAKEN IF GROSSLY INVOLVED BY ENDOMETRIAL CARCINOMA?

### Recommendation

At least 2 representative sections of tumor involving the cervix should be submitted. These sections must include the full thickness of the cervical wall and the ectocervical or vaginal cuff margin.

Documenting involvement of the cervical stroma ensures accurate staging (FIGO stage II) 39. In addition, providing the depth of cervical stromal invasion in relation to the full thickness of the cervical wall might be required for adjuvant therapy purposes. The following guidelines are used by some radiation oncology groups: (1) if <3 mm cervical stromal invasion and no other feature suggesting the need for additional adjuvant pelvic irradiation, patients are treated with vaginal brachytherapy only; (2) if invasion into the outer one-third of the cervical stroma or invasion into the middle one-third with vascular/lymphatic invasion, adjuvant pelvic radiation is recommended; (3) if no evaluation of lymph nodes was performed at time of surgery, pelvic radiation is recommended for most patients who have >2 to 3 mm of cervical stromal invasion, particularly if the tumor is high grade 12.

## HOW SHOULD A MORCELLATED HYSTERECTOMY SPECIMEN THAT CONTAINS AN UNEXPECTED ATYPICAL ENDOMETRIAL HYPERPLASIA/ EIN OR ENDOMETRIAL CARCINOMA BE HANDLED?

### Recommendation

Gross examination of a morcellated hysterectomy specimen requires special attention to identify any endometrial abnormality, although this may be extremely difficult to see in a morcellated specimen. If such an abnormality is detected, the entire endometrial lesion and the adjacent myometrium should be submitted for microscopic examination. In addition, sampling of myometrial tissue containing any serosal surface should be undertaken. If the endometrium appears grossly unremarkable and the initial representative sections demonstrate the presence of atypical endometrial hyperplasia/EIN or endometrial carcinoma, careful regrossing is required with the submission of all the visible endometrial lining and adjacent myometrium. If the morcellated specimen contains the uterine cervix, this should be sampled representatively.

Unexpected endometrial cancer in morcellated hysterectomy specimens obtained for presumptive benign conditions is uncommon with a reported incidence ranging from 0.07% to 3% 67–75. In general, it is recommended that in such cases a preoperative endometrial biopsy is performed to exclude such a possibility. Although the use of endometrial dye instillation, either trypan blue or methylene blue 76,77, has been proposed as a useful technique to facilitate the identification of the endometrium in morcellated hysterectomies, this is not commonly done. When an occult cancer is picked up in a morcellated specimen, it may be very difficult to assess the depth of myoinvasion and thus accurate staging of the endometrial cancer may not be possible 78.

## HOW THOROUGHLY SHOULD THE FALLOPIAN TUBES BE EXAMINED?

### Recommendation

Gross examination of the fallopian tube must be carefully undertaken and any areas with macroscopic abnormalities should be submitted for microscopic examination. If the fallopian tube is unremarkable, the entire tube should be submitted for microscopic examination using the SEE-FIM protocol. Should this not be possible in all cases of endometrial cancer, the SEE-FIM protocol should be used if uterine serous carcinoma, clear cell carcinoma and carcinosarcoma are present, while only the fimbrial end should be submitted in toto in other scenarios—using the guidelines of the SEE-FIM protocol, along representative cross-sections of the remainder of the fallopian tube.

**FIG. 7:**
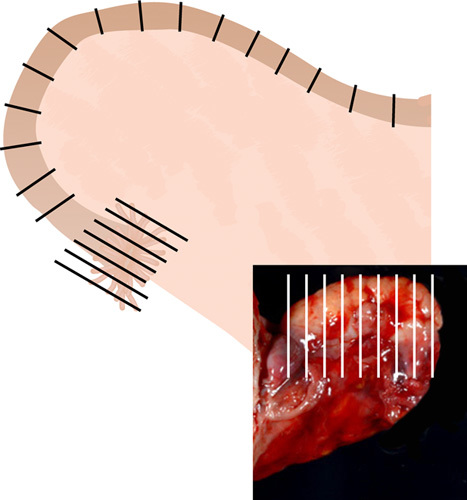
Sectioning and Extensively Examining the FIMbriated End protocol, sectioning of adnexa to be submitted for microscopic examination.

The SEE-FIM protocol encompasses the following steps: (1) fixing the specimen for several hours, (2) amputating the distal 2 cm—the infundibulum and fimbrial end, and sectioning parallel to the long axis of the fallopian tube, (3) taking cross sections at 2 to 3 mm intervals of the rest of the tube—the isthmus and ampulla, (4) sectioning the ovary perpendicularly to its long axis at 2 to 3 mm intervals and (5) to examine one hematoxylin and eosin (H&E)-stained slide per block 7 (Fig. 7). Although this system is being used for all endometrial cancers at some institutions 79, and its universal use in endometrial cancer is advocated by some investigators 80,81, at this point there is not enough evidence to support its mandatory application regardless of histologic type 82. Of note, it has been stated that expenses secondary to complete processing are relatively minor when balanced against potential expenses of a recurrence 82. A practical alternative is to ensure that at least the fimbrial end of the fallopian tube, as per the SEE-FIM protocol, is examined in addition to the usual representative sections of the rest of the fallopian tube 28,83.

## HOW MUCH OF A NORMALLY SIZED AND GROSSLY UNREMARKABLE OVARY SHOULD BE SUBMITTED?

### Recommendation

Gross examination of the ovary must be carefully performed. If serous carcinoma, clear cell carcinoma or carcinosarcoma, the entire ovary should be submitted after slicing it perpendicularly to its long axis at 2 to 3 mm intervals. If possible, the same protocol should be used for oophorectomy specimens accompanying hysterectomies for other endometrial cancer histotypes. Should the latter not be possible, at least 2 sections of each ovary should be submitted.

Some investigators advocate the universal use of the SEE-FIM protocol to handle grossly unremarkable adnexa for endometrial cancer 79,81. Others proposed that regardless of histotype, the ovaries should be submitted in toto if they are small and unremarkable; should they not be small, at least 2 sections should be submitted 84. Of note, one study found that 2.7% of grossly unremarkable ovaries removed as part of the surgical treatment for endometrial cancer harbor microscopic carcinoma 82.

## HOW MANY SECTIONS OF OMENTUM SHOULD BE EXAMINED HISTOLOGICALLY WHEN REMOVED FOR STAGING PURPOSES IN CASES OF SOME ENDOMETRIAL CARCINOMA HISTOTYPES?

### Recommendation

Omentectomy is part of the staging procedure of endometrial serous carcinoma, clear cell carcinoma and carcinosarcoma. The gross appearance and measurement of the omentum should be provided. Omental tissue should be sliced at 0.5 cm intervals to detect small abnormalities. There are no standard sampling recommendations, but in general the number of sections to be submitted will depend on the gross examination findings. If the omentum is grossly positive, one or 2 representative sections are enough for microscopic evaluation, but if it is grossly negative, one representative section per 2 or 3 cm of maximal omental dimension 85 or at least a total of 4 blocks of tissue should be submitted 28.

Currently, guidelines on omental tissue sampling in endometrial cancer are included in the Dataset for Histological Reporting of Endometrial Cancer by the Royal College of Pathologists 28, but not included in the recommendations of either the CAP 11 or the International Collaboration on Cancer Reporting 86 The former recommends submission of a total of 4 blocks of tissue if grossly negative omentum. Others, advocate for submission of one representative section per 2 or 3 cm of maximal omental dimension if the omentum is grossly unremarkable 73. One study found that submitting 5 blocks of grossly negative omentum has a sensitivity of 82% while examining 10 blocks raises the sensitivity to 95% 87.

## HANDLING OF LYMPH NODES

### Recommendations

Lymph nodes from different anatomical sites should be sent in separate appropriately labelled specimen containers and handled separately. They should be carefully dissected from the adipose tissue. This can be done with a thorough visual examination and palpation. Clearing solutions are not routinely recommended as they are not usually necessary. A small amount of adipose tissue should be left around larger lymph nodes to evaluate the presence or absence of extranodal extension. Lymph nodes up to 2 mm are embedded whole. If larger than 2 mm, they should be sliced in neat, parallel slices at 2 to 3 mm intervals—slicing should be perpendicular to the long axis of the node. All grossly unremarkable lymph node tissue should be submitted for microscopic examination in properly identified cassettes. The number of lymph nodes submitted per cassette and the way they have been submitted, for example in toto—if very small, or sectioned, should be specified in the section code. With grossly positive lymph nodes, representative sections to demonstrate the largest size of tumor involvement as well as the surrounding adipose tissue should be submitted for microscopic examination and noted in the section code.

Nodal involvement is one of the most powerful prognostic determinants in all cancers, and it predicts distant recurrences in low-risk endometrial carcinoma 88. At the same time systematic pelvic (+/− para-aortic) nodal dissection, which is associated with significant morbidity, has demonstrated no survival benefit 89,90. and practices vary worldwide for this reason. The proper retrieval of lymph nodes can be achieved with a thorough examination by direct vision, palpation, and sharp dissection and does not require the use of clearing solutions 91. Slicing lymph nodes perpendicular to their long axis at 2 mm intervals increases the chance of detecting metastases 92,93. Submitting a rim of adipose tissue around the lymph nodes allow the assessment of extracapsular extension when there is tumor involvement. The latter finding has been reported to be an important prognostic factor in FIGO stage IIIC endometrial carcinoma by some investigators 94.

## HANDLING OF SLNS

### Recommendations

**FIG. 8:**
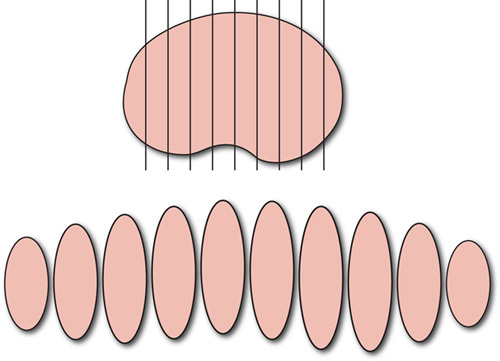
Sentinel lymph node, parallel slices are perpendicular to the long axis of the specimen.

The description of the SLN should include measurements, gross appearance, the presence of dye and the radioactive tracer reading provided by the surgeon, if any. The lymph node is sliced at 2.0 mm intervals perpendicular to its long axis (Fig. 8). A small rim of adipose tissue should be left around the lymph node. The entire lymph node is submitted for microscopic examination in properly identified cassettes. The SLN is usually ultrastaged [i.e. additional recuts and/or immunohistochemistry (IHC) for keratin], although some institutions do not routinely undertake ultrastaging. At the present time there is no universal ultrastaging protocol; however, all institutions undertaking SLN examination should have a standard procedure for SLNs in endometrial cancer.

SLN removal has been introduced in the surgical staging of endometrial carcinoma to decrease the morbidity secondary to a lymphadenectomy but still obtain information about the lymph node status 95. The 2018 NCCN Guidelines indicate that SLN mapping may be considered in patients with apparent uterine-confined endometrial cancer (clinical stage I disease) 57. As delineated in the NCCN guidelines and the Society of Gynecologic Oncology recommendations, there are several key points when using this technique: (1) the expertise of the surgeon and attention to technical detail are critical to ensure mapping success; (2) superficial and deep cervical injection of dye has been validated as a useful mapping technique; (3) complete evaluation of the peritoneal cavity is mandatory; (4) SLN dissection starts with the evaluation of the retroperitoneal spaces and identification of the sentinel drainage pathways that emanate from the parametrial tissue, this is followed by excision of all mapped SLNs; (5) any suspicious non-SLN should be excised and frozen section may be required to determine if a para-aortic lymphadenectomy will be performed. Of note, routine frozen section of SLNs is not advisable as small foci of tumor may be lost when cutting the frozen section slides in addition to the relatively low sensitivity for detection of metastases in grossly unremarkable lymph nodes, (6) should mapping failure occur in a hemi-pelvis, a side-specific lymphadenectomy should be performed; (7) pathology ultrastaging is required to improve the detection of low-volume metastases 57,95. The 2018 NCCN guidelines also state that recent evidence indicates that SLN mapping may be used in high-risk histologies (serous carcinoma, clear cell carcinoma, and carcinosarcoma) 57. At the present time, it has been established that the use of indocyanine green, which requires use of a near infra-red camera for localization, has similar rates of mapping success to those of radiocolloid Tc-99 combined with blue dye 95. The gross processing of SLNs is critical to ensure the success of this technique. It is of utmost importance to obtain serial perpendicular, thin (2.0 mm) sections as an initial step as this raises the odds of metastatic tumor detection independent of the ultrastaging process 84. This method not only facilitates the examination of the lymph node subcapsular space and parenchymal surface, but is designed to detect all metastases >2.0 mm. A study comparing 2 different ultrastaging protocols (method #1, obtaining 5 H&E-stained levels at 250 μm with 2 unstained slides at each level—pankeratin IHC performed on level 1 in cases with negative H&E or method #2, 1 H&E level and 2 unstained slides cut at 250 μm into the tissue block, pankeratin IHC performed in cases with negative H&E) found no statistically significant differences between the methods with respect to number of positive SLNs detected, size of metastasis or false-negative rate 84.

There is no universal protocol for the ultrastaging of SLNs 95. Protocols used at the 2 largest cancer centers in the United Stated are as follows:

1. The University of Texas M.D. Anderson Cancer Center Protocol.

**FIG. 9:**
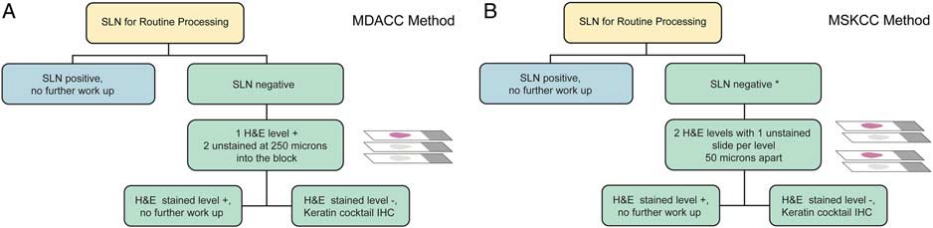
Ultrastaging protocols, MD Anderson Cancer Center (MDACC) (A) and Memorial Sloan Kettering Cancer Center (MSKCC), ultrastaging will be obtained if there is myometrial or vascular/lymphatic invasion (*) (B). H&E indicates hematoxylin and eosin; IHC, immunohistochemistry; SLN, sentinel lymph node.

If the H&E-stained slide is negative for tumor, 3 consecutive sections at 250 µm into the paraffin block are obtained (one for H&E and one of the remaining 2 to be used for keratin cocktail IHC if the additional H&E-stained slide is negative 96 (Fig. 9A).

1. Memorial Sloan Kettering Cancer Center Protocol.

If the initial H&E-stained slide is negative for carcinoma and the endometrial cancer is myoinvasive or associated with vascular/lymphatic invasion, 2 additional levels 50 µm apart are examined, at each level 2 slides are obtained, one for H&E and the second for keratin cocktail IHC if the H&E-stained slide is negative 97 (Fig. 9B).

## REPORTING MARGINS OF HYSTERECTOMY SPECIMENS FOR ENDOMETRIAL CANCER

### Recommendations

The ectocervical margin of a hysterectomy specimen should be reported in an endometrial cancer with cervical involvement. The vaginal cuff and parametrial margins should be reported in endometrial carcinomas with cervical and/or parametrial involvement when a radical hysterectomy is undertaken. Otherwise, reporting of margins is optional 11. In cases where it is required to report the above margins, including the distance between the tumor and the margin is also optional 11. Involvement of the uterine serosa by tumor should be reported as this finding indicates FIGO stage III A disease 39; however, the uterine serosa is not a margin and should not be designated as such.

## INTRAOPERATIVE ASSESSMENT

### Recommendations

In cases where intraoperative assessment is requested by the surgeon to determine whether staging will be obtained, examine the specimen carefully, including the uterine serosa, lower uterine segment, cervix and adnexal structures. Identify the lesion, measure it, cross-section the uterine wall, and evaluate the status of the myometrium. Submit at least one section of the lesion for frozen section including the adjacent uterine wall. Should myometrial invasion be suspected, submit the lesion and the full thickness of the uterine wall containing the deepest point of myometrial invasion for frozen section. Should cervical, uterine serosal or adnexal involvement be suspected, submit appropriate sections for frozen section examination.

Intraoperative evaluation of hysterectomy and bilateral salpingo-oophorectomy specimens obtained for endometrial cancer to determine parameters that guide lymph nodes dissection, and in some cases omentectomy, which are needed for staging and prognosis, is commonly performed in the United States, and much less frequently, if at all, in other parts of the world 31. Histotype, tumor grade if applicable, and depth of myometrial invasion are typically reported 31. In addition, tumor size needs to be determined if the Mayo algorithm is being applied. Briefly, using this algorithm cases are stratified as low risk or high risk. Low risk is defined as FIGO grade 1 or 2 endometrioid carcinoma with myometrial invasion ≤50% and primary tumor diameter ≤2 cm while high risk is defined as FIGO grade 3 endometrioid carcinoma, nonendometrioid histotype, myometrial invasion >50% or primary tumor diameter >2 cm. Cases in the low risk category are spared a systematic pelvic and para-aortic lymphadenectomy while these procedures are performed in cases in the high risk category 21. Lower uterine segment, cervical, and adnexal involvement and lymphovascular space invasion should be reported if these findings are identified during the intraoperative evaluation.

## CONCLUSIONS

It is our hope that these ISGyP developed recommendations will help to standardize the processing of endometrial cancer specimens; this will facilitate accurate pathologic reporting and a better understanding of this disease which will be to the ultimate benefit of patients suffering from it.
